# Supplementary figures and images for: An extended catalogue of tandem alternative splice sites in human tissue transcriptomes
Source: PLoS Comput Biol. 2021 Apr 7;17(4):e1008329. doi: 10.1371/journal.pcbi.1008329 (PMC8055015; doi:10.1371/journal.pcbi.1008329)

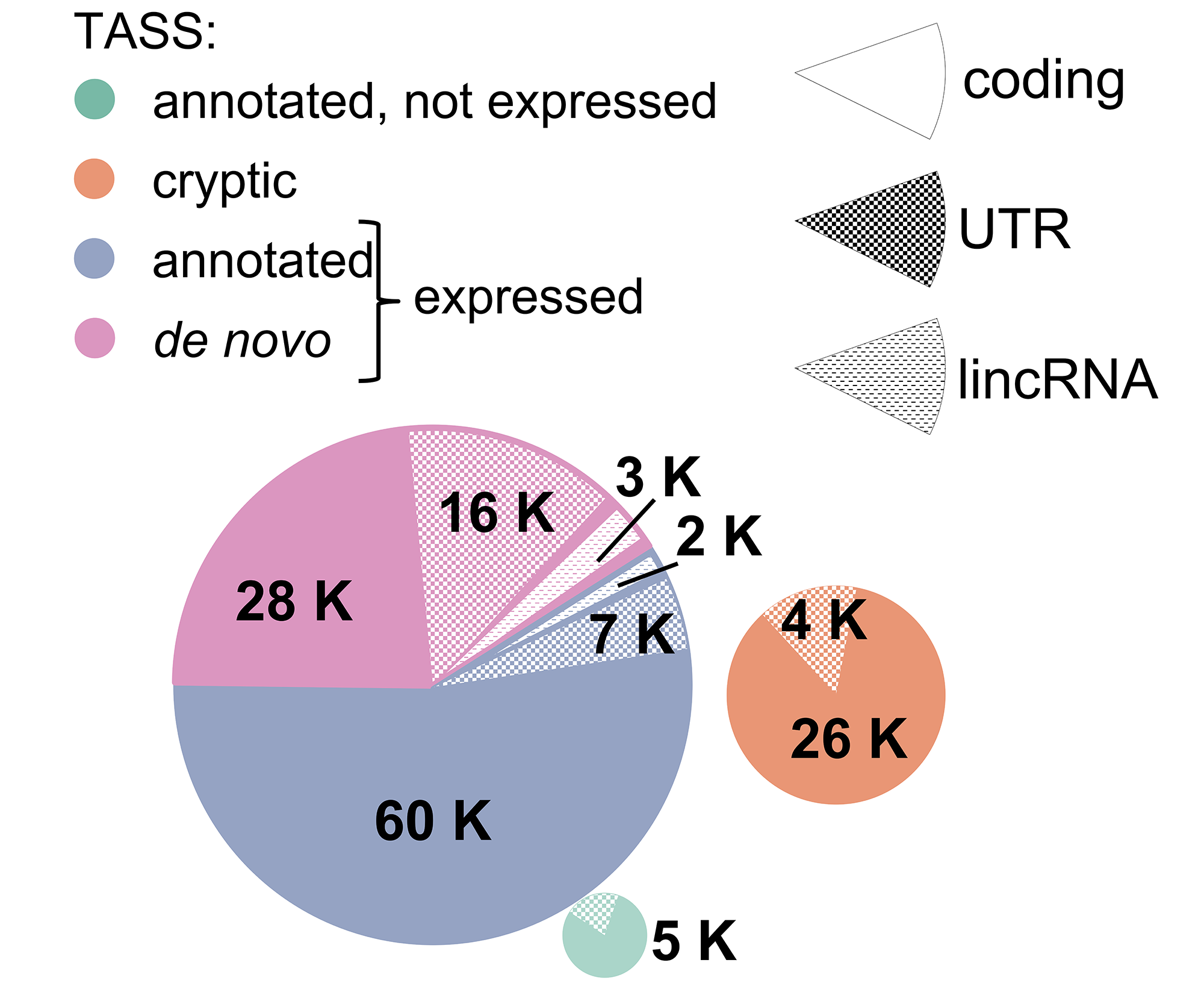

Supplement: S1 Fig — In addition to the numbers provided in the figure, there are 163 cryptic splice sites in lincRNA genes and 580 annotated but not expressed splice sites in lincRNA genes. (TIF) [file pcbi.1008329.s001.tif]

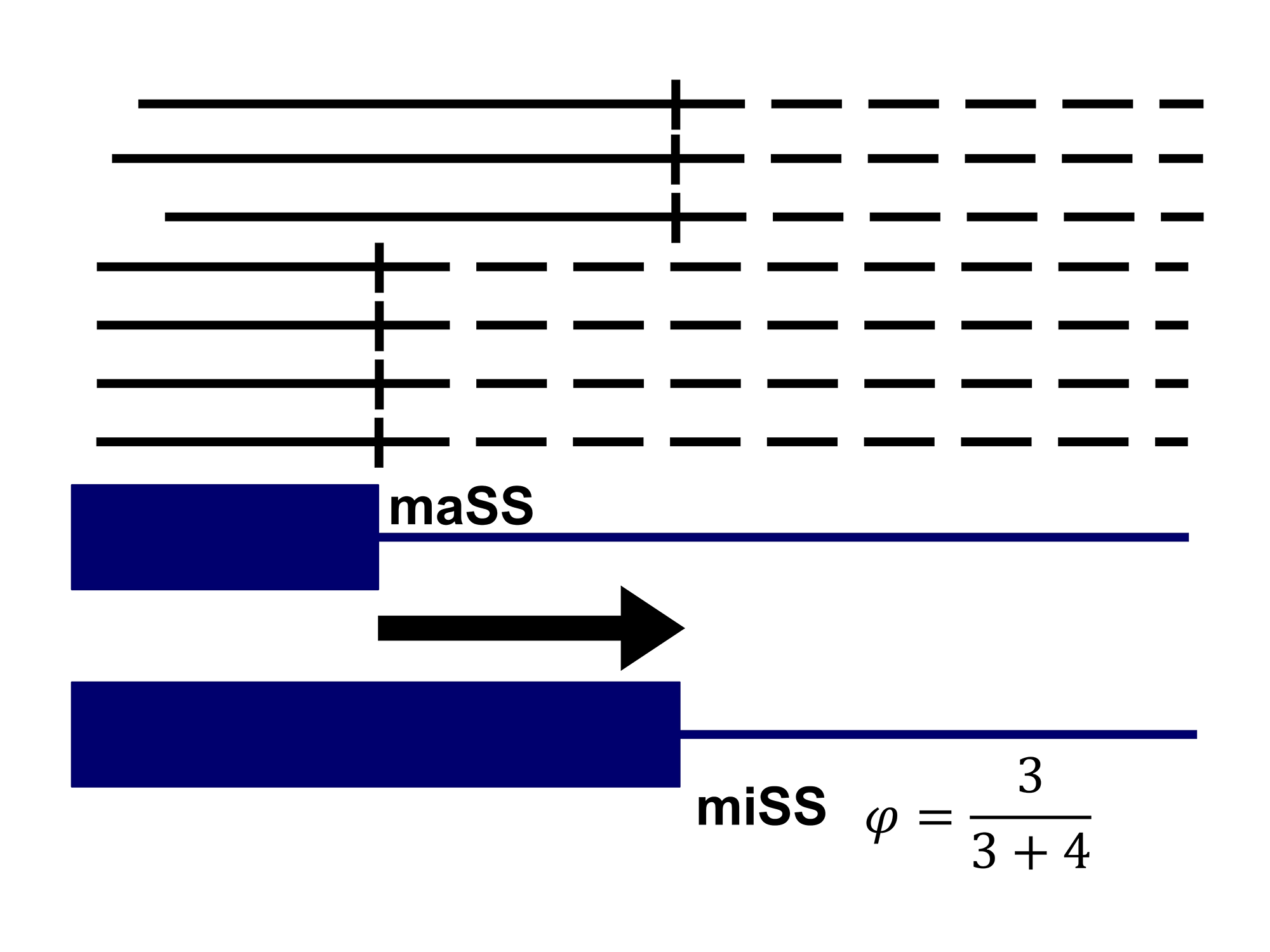

Supplement: S2 Fig — A hypothetical maSS is supported by 4 split reads, while a hypothetical miSS is supported by 3 split reads, resulting in the φ value of 3/7. (TIF) [file pcbi.1008329.s002.tif]

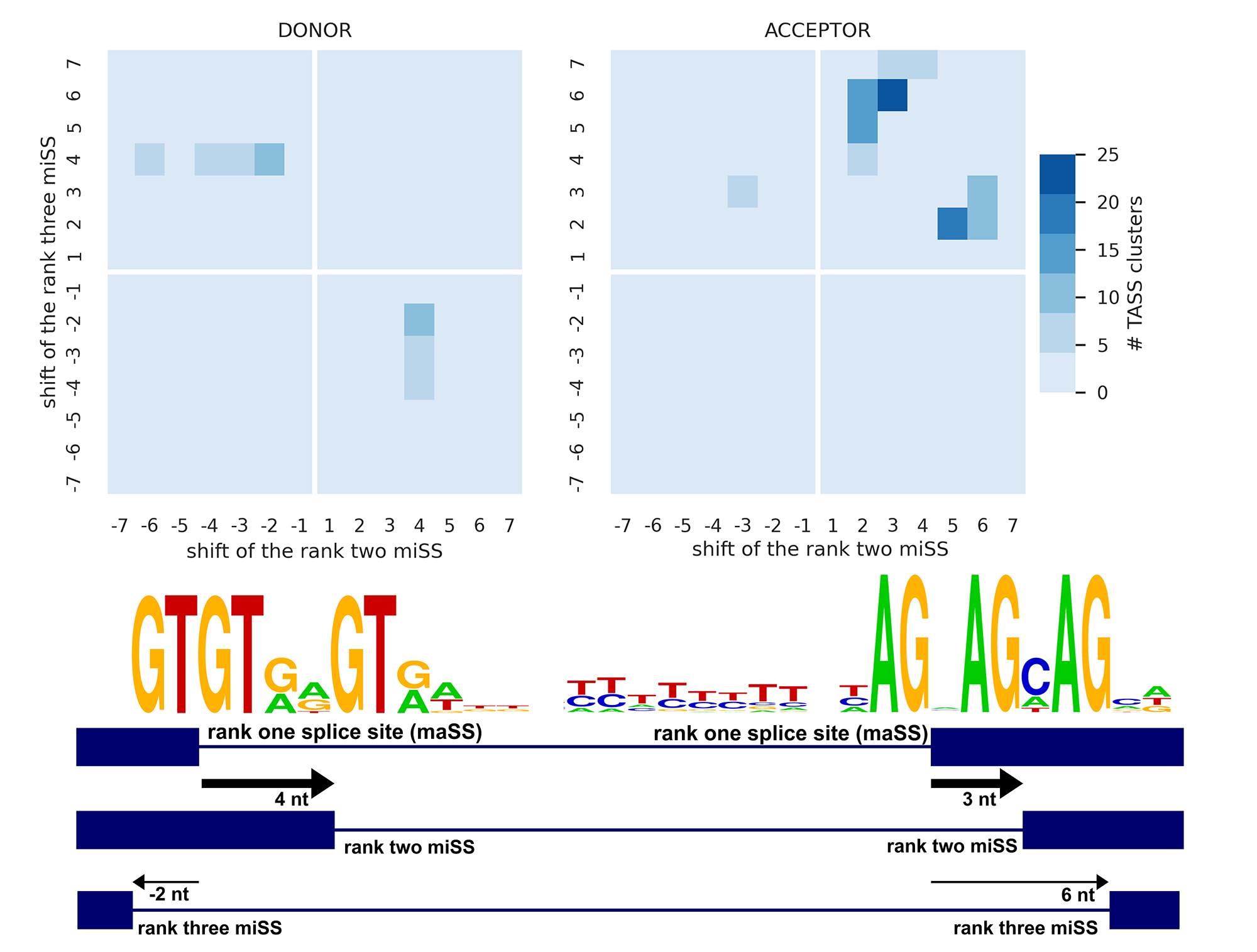

Supplement: S3 Fig — A TASS cluster of size three is characterized by two shift values: the rank two miSS relative to maSS, and rank three miSS relative to maSS. The top panel shows the joint distribution of rank two miSS shift (x-axis) and rank three miSS shift (y-axis) for donors (left) and (acceptors). The bottom panel shows LOGO charts of miSS sequences corresponding to shifts of +4 and -2 for the donor splice site, and +3 and +6 shifts for the acceptor splice site. (TIF) [file pcbi.1008329.s003.tif]

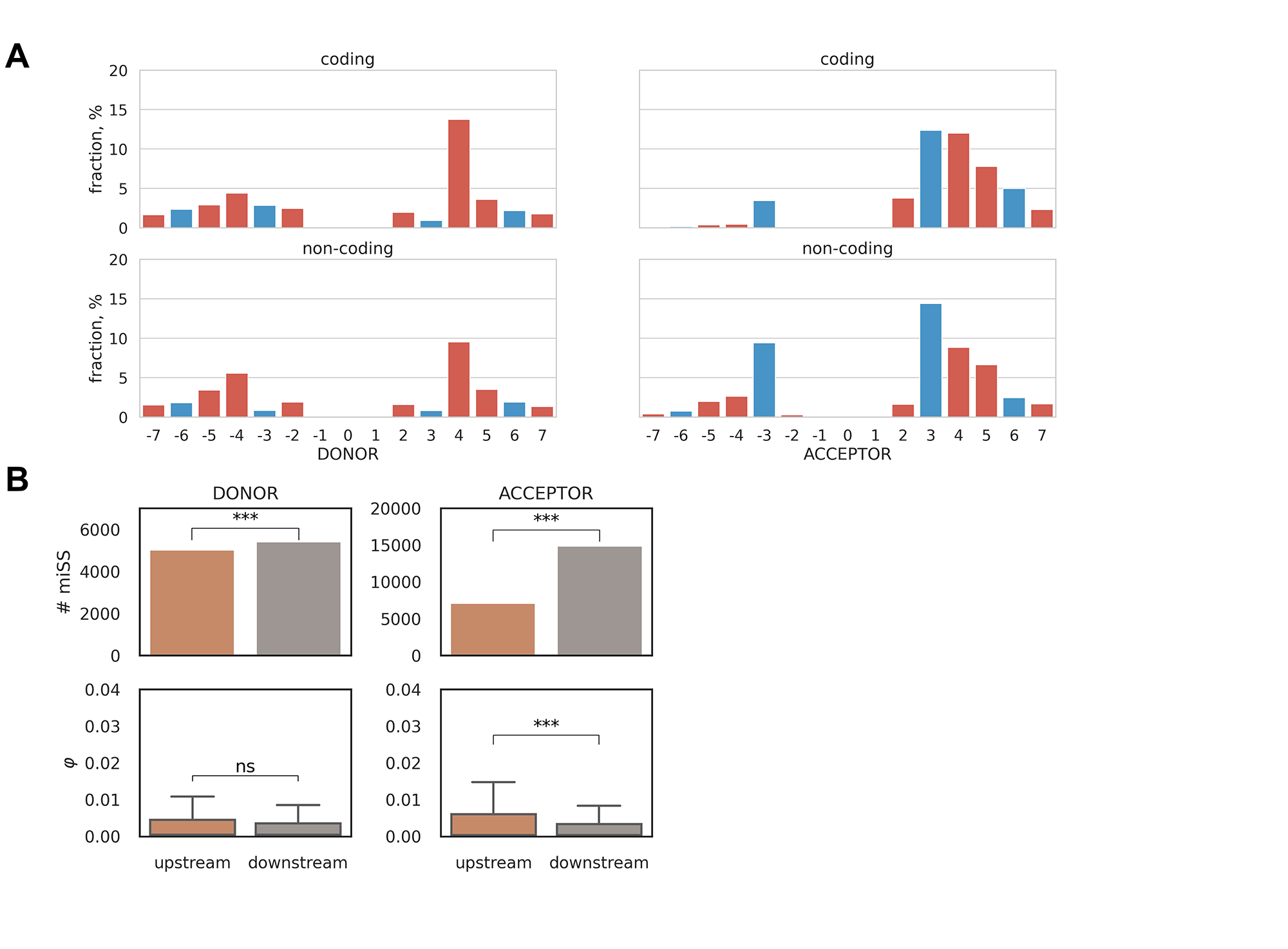

Supplement: S4 Fig — (A) Shift frequencies in coding vs. non-coding regions (see Fig 1G for comparison). (B) The abundance and relative expression of upstream vs. downstream shifts. (TIF) [file pcbi.1008329.s004.tif]

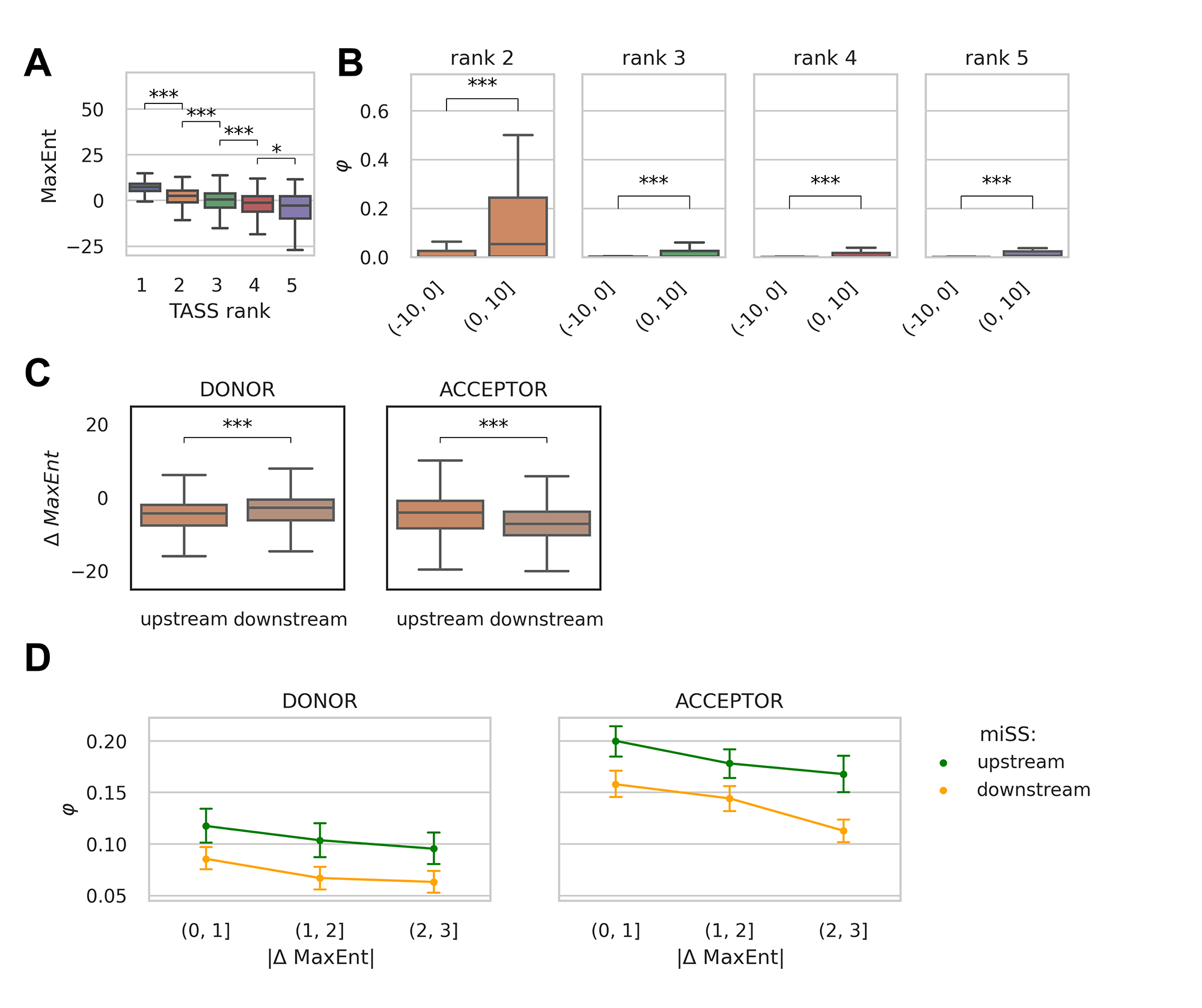

Supplement: S5 Fig — (A) According to MaxEnt scores, maSS (i.e., rank one sites) are on average stronger than miSS (i.e., rank 2,3,4,5). (B) Within each rank group, the relative usage of a miSS (φ) generally increases with increasing Δ MaxEnt value, its strength relative to that of the maSS. (C) The distribution of Δ MaxEnt values for upstream and downstream shifts. (D) The relative usage of a miSS (φ) as a function of the absolute difference of TASS strengths. The upstream miSS are used more frequently when the splice sites are nearly of the same strength. (TIF) [file pcbi.1008329.s005.tif]

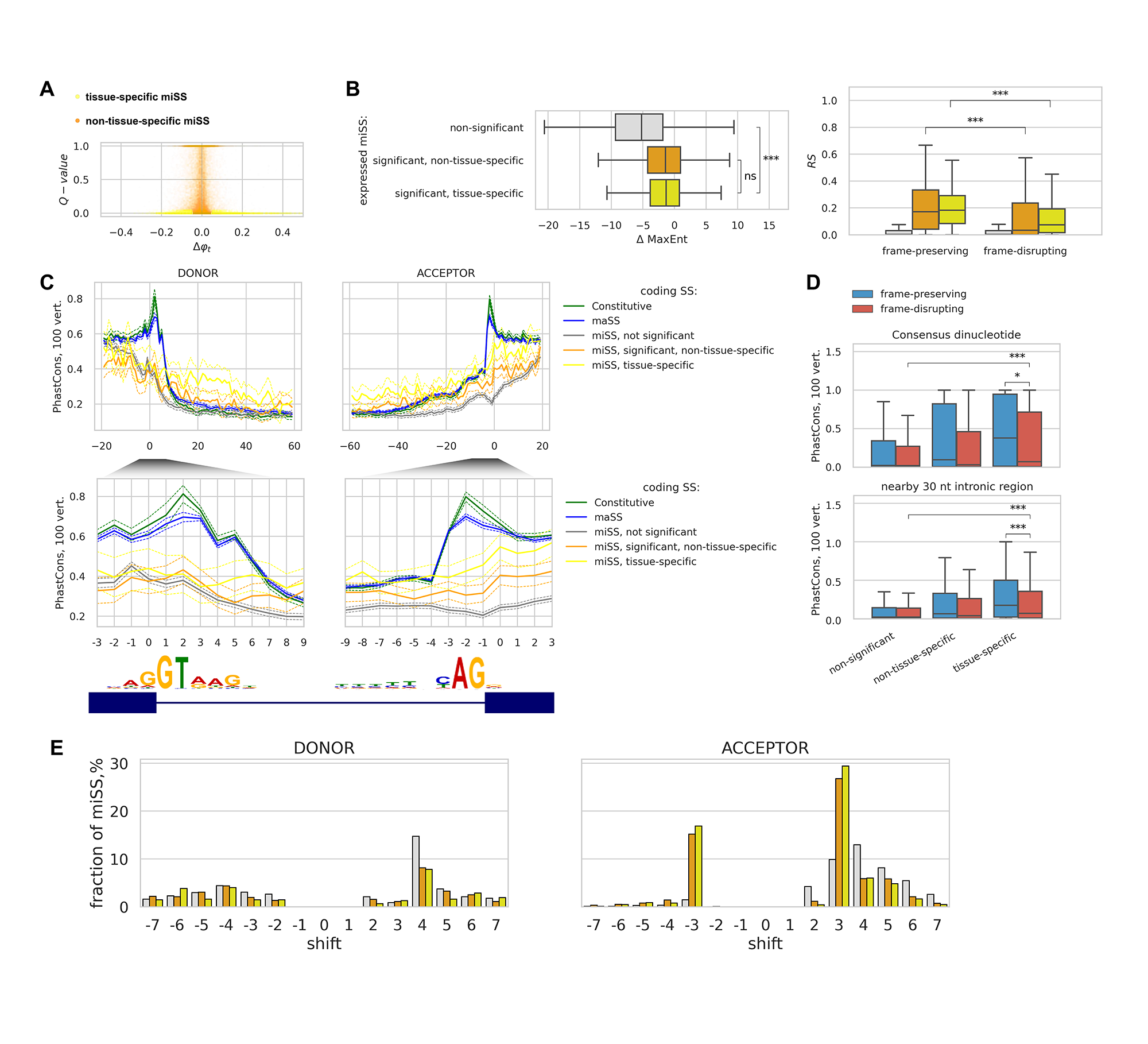

Supplement: S6 Fig — (A) Tissue-specific miSS are defined to have |Δφt|>0.05 (x-axis) and Q-value <0.05 (y-axis) in at least one tissue. (B) The distribution of Δ MaxEnt values for miSS in different expression categories (left). The distribution of RS (RiboSeq support) values for miSS of different expression categories in protein-coding regions (right). (C) The average PhastCons scores (100 vertebrates) for positions near the miSS in different expression categories. (D) The distribution of average PhastCons scores (100 vertebrates) at the consensus dinucleotides of splice sites (top) and average PhastCons scores of the adjacent 30 nt intronic regions (bottom). (E) The distribution of shifts for non-significant, non-tissue-specific and tissue-specific donor and acceptor miSS. (TIF) [file pcbi.1008329.s006.tif]

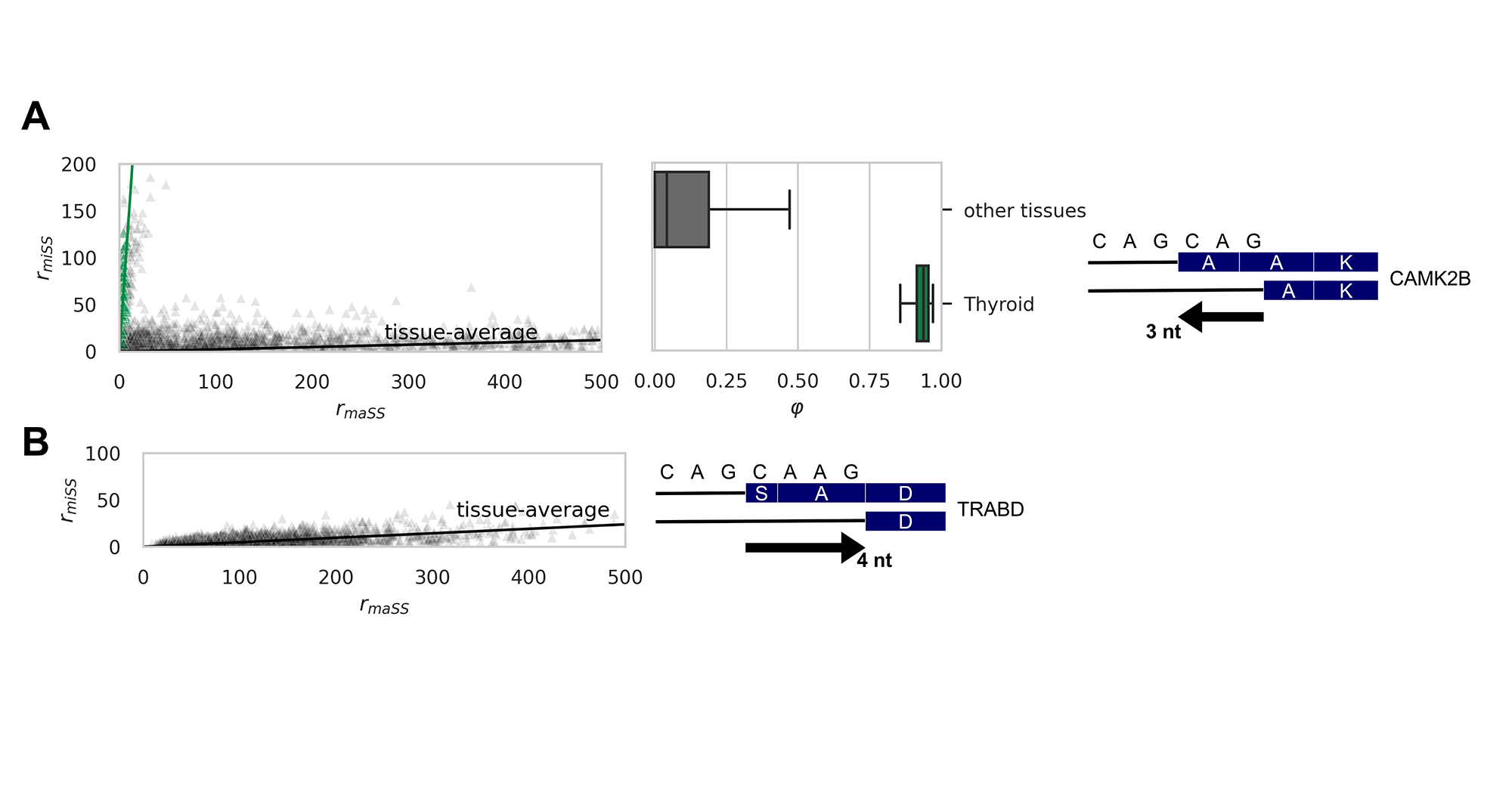

Supplement: S7 Fig — (A) A thyroid-specific miSS in exon 14 of the gene CAMK2B. The miSS becomes a maSS in thyroid as its φ value exceeds 0.5. (B) The miSS in exon 8 of the TRABD gene is non-tissue-specific. (TIF) [file pcbi.1008329.s007.tif]

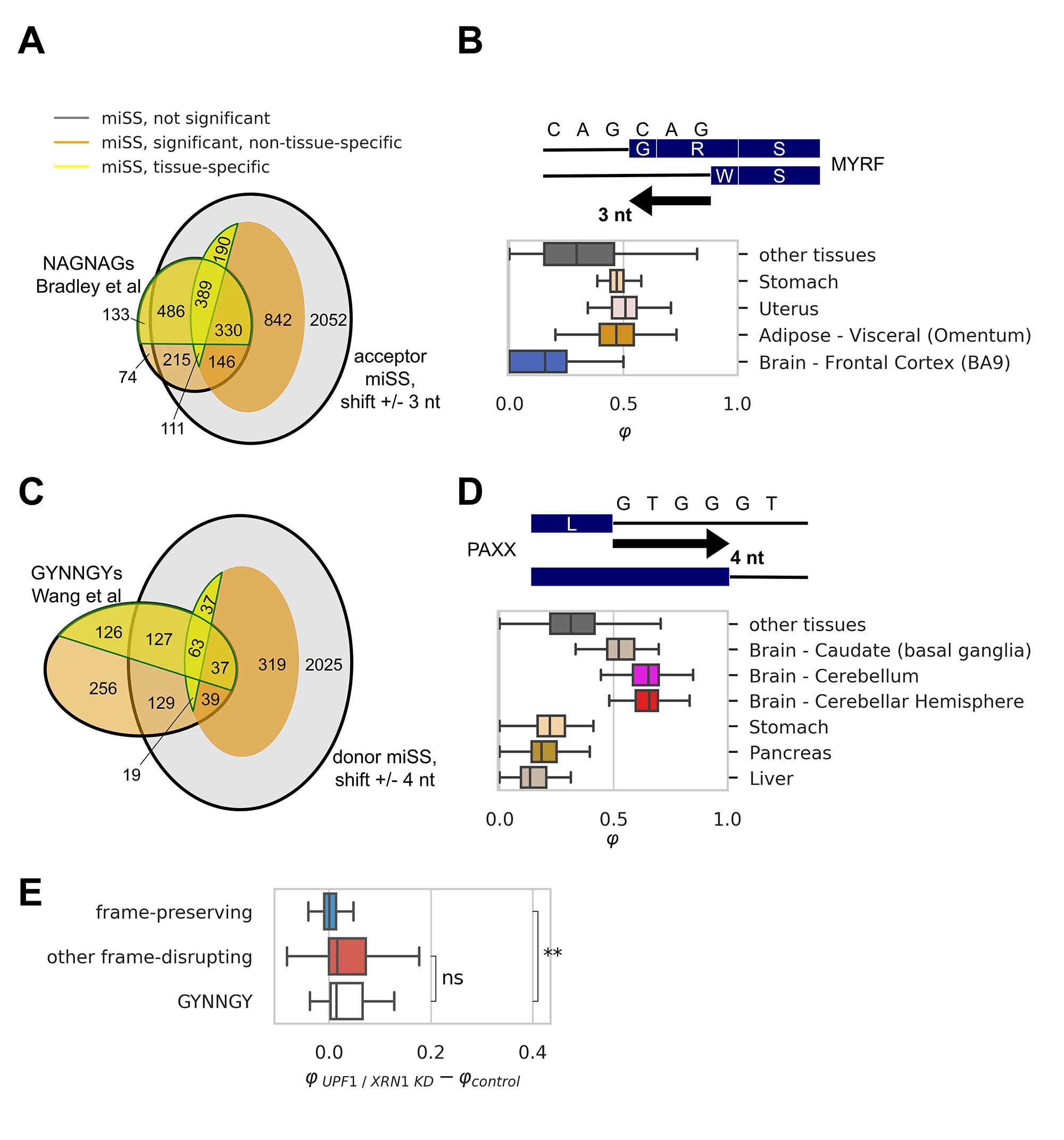

Supplement: S8 Fig — (A) The intersection of the acceptor miSS located ±3 nts from the maSS with the list of NAGNAGs provided by Bradley et al [5]. (B) A NAGNAG acceptor splice site in the exon 20 of the MYRF gene. The upstream NAG is upregulated in the stomach, uterus, adipose tissues and downregulated in the brain. (C) The intersection of the donor miSS located ±4 nts from maSS with the list of GYNNGYs provided by Wang et al [1]. (D) A GYNNGY donor splice site in the exon 2 of the PAXX gene. The downstream GY is upregulated in the brain and downregulated in the stomach, pancreas, and liver tissues. (E) The response of GYNNGY miSS to NMD inactivation. (TIF) [file pcbi.1008329.s008.tif]

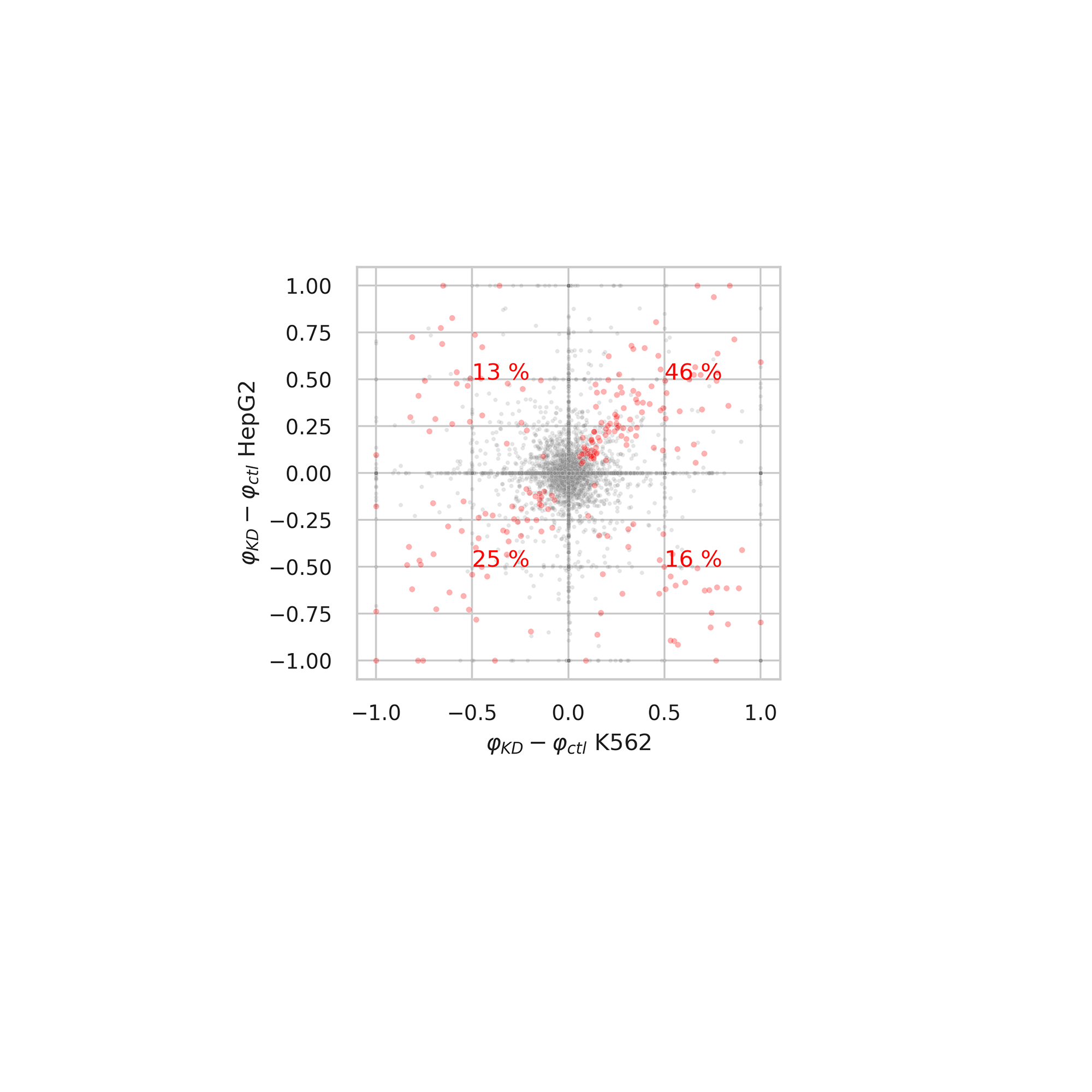

Supplement: S9 Fig — Fractions of significant miSS-RBP pairs located in each quadrant are shown (the fractions are summed to 100%). (TIF) [file pcbi.1008329.s009.tif]

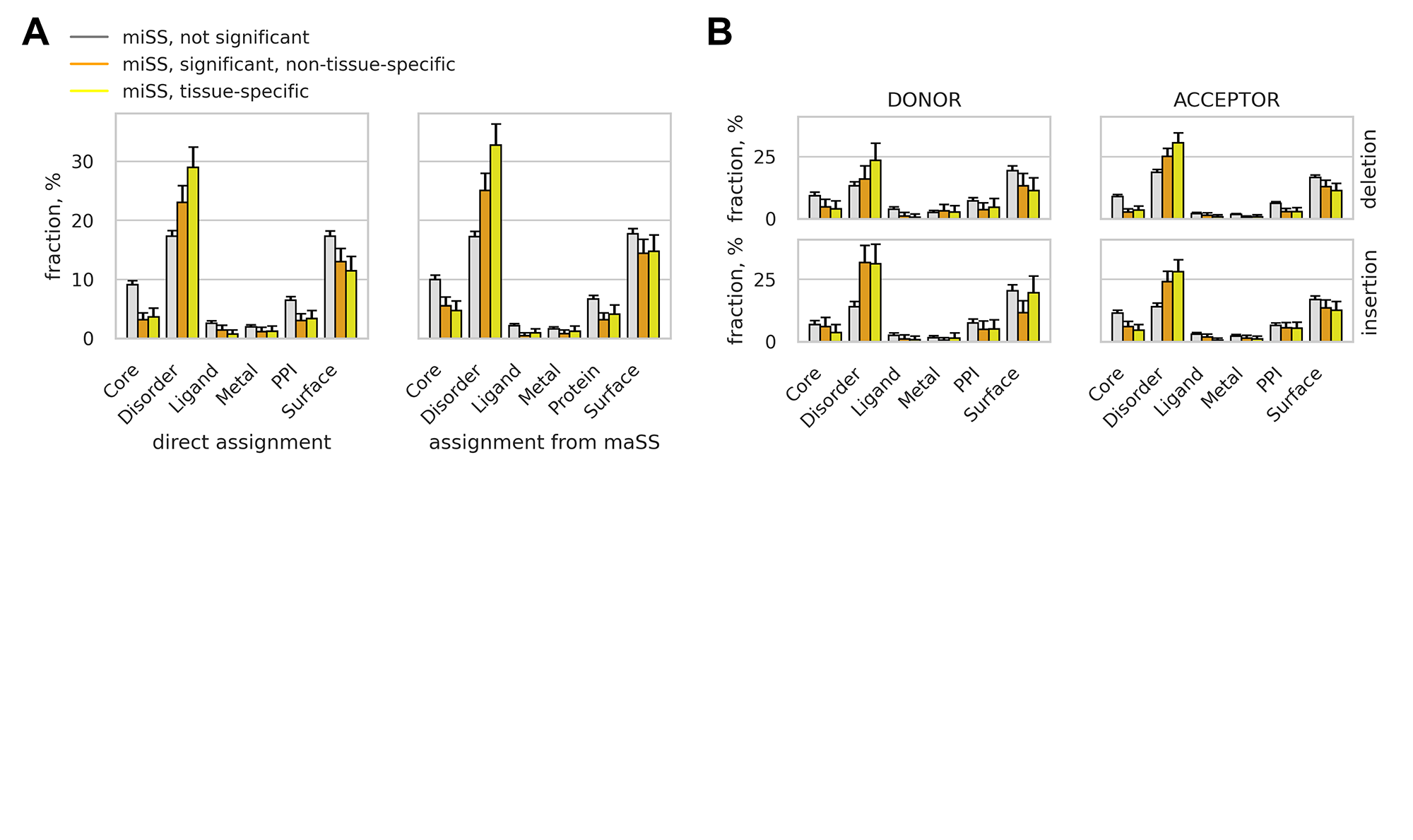

Supplement: S10 Fig — (A) The comparison of the structural annotation assigned directly to miSS (left) or from the structural annotation of the corresponding maSS (right). Only exonic miSS and corresponding maSS are considered. (B) The structural annotation for different categories of miSS. (TIF) [file pcbi.1008329.s010.tif]

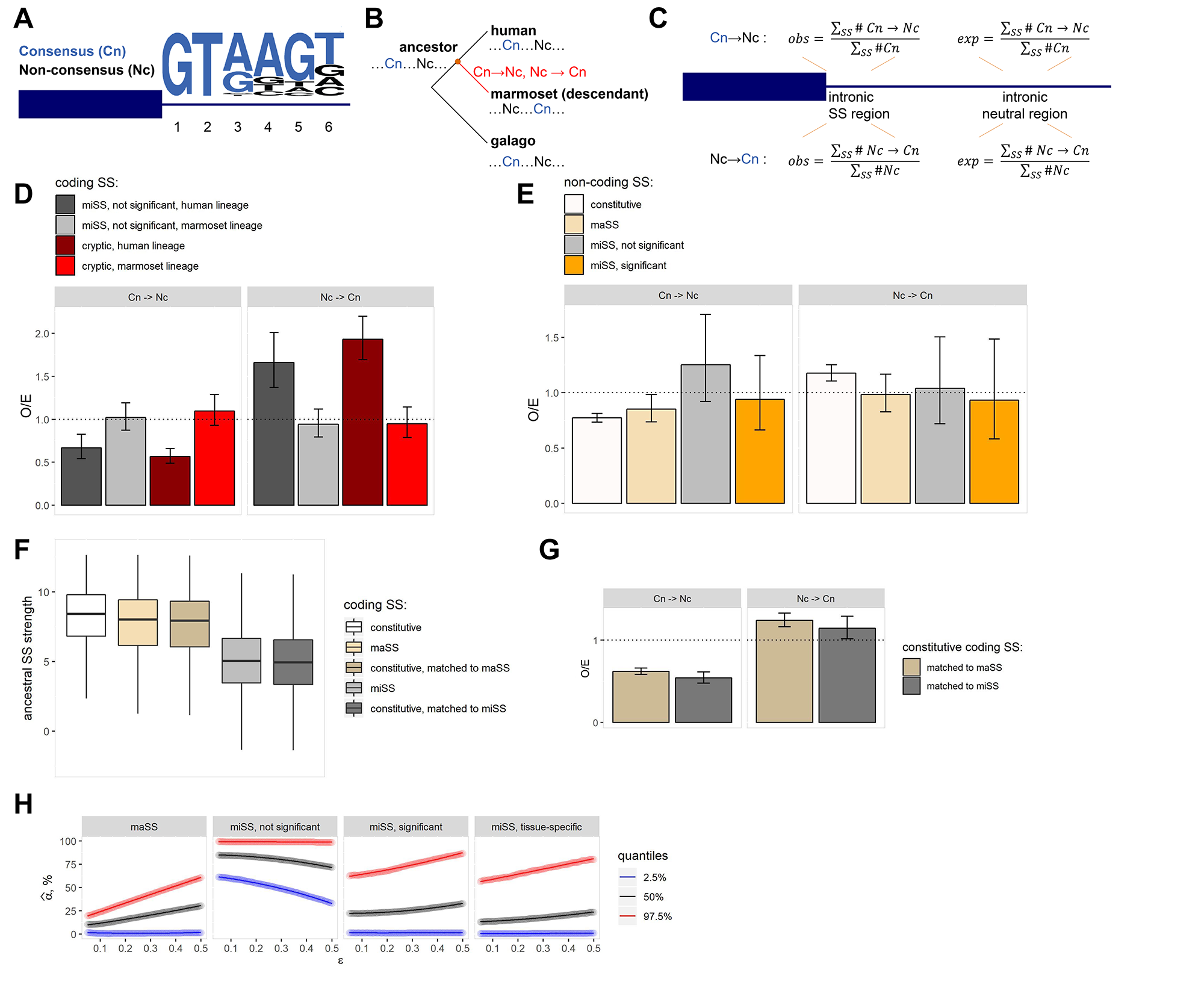

Supplement: S11 Fig — (A) The definition of the consensus (Cn) and non-consensus (Nc) nucleotide variants in the donor splice site. The definition for acceptor splice site is similar. (B) The evolutionary tree used to reconstruct the ancestral sequence of human and marmoset. (C) The computation of obs and exp statistics. (D) The selection of cryptic and not significant miSS in coding regions for marmoset and human genomes. (E) The strength of the selection in selected categories of splice sites in the non-coding regions (F) The distribution of ancestral strength for different splice site categories. (G) The strength of the selection acting on constitutive coding splice sites matched to miSS and maSS by the ancestral splice site strengths. (H) Estimation of the 95% confidence interval of α for different expression categories of miSS. (TIF) [file pcbi.1008329.s011.tif]

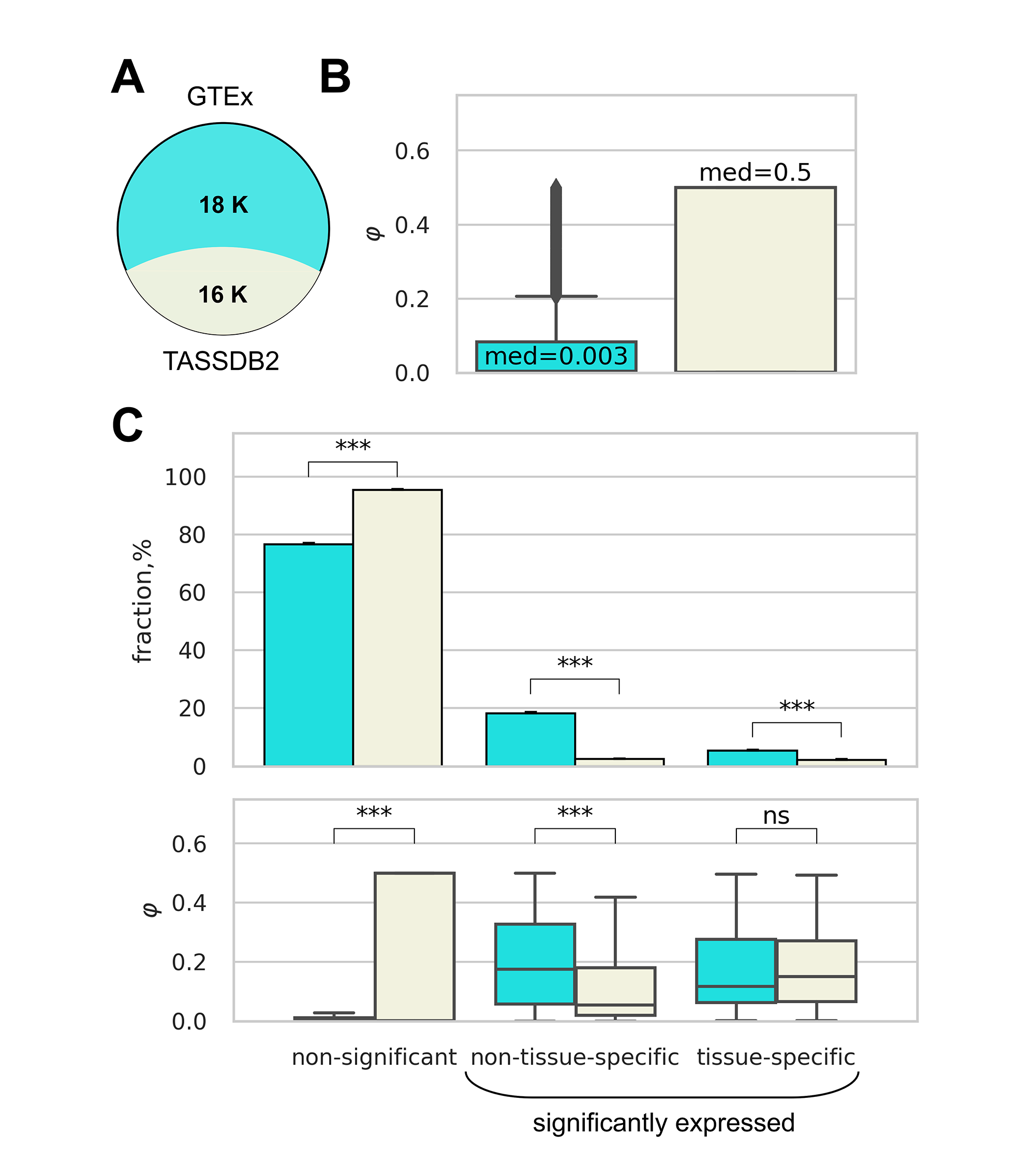

Supplement: S12 Fig — (A) The intersection of the set of expressed miSS with TASSDB2. (B) miSS not contained in TASSDB2 have on average lower φ values than miSS in TASSDB2. (C) miSS not contained in TASSDB2 are enriched with tissue-specific and non-tissue-specific significantly expressed miSS (top); within these categories they have similar or higher φ values compared with miSS in TASSDB2 (bottom). (TIF) [file pcbi.1008329.s012.tif]

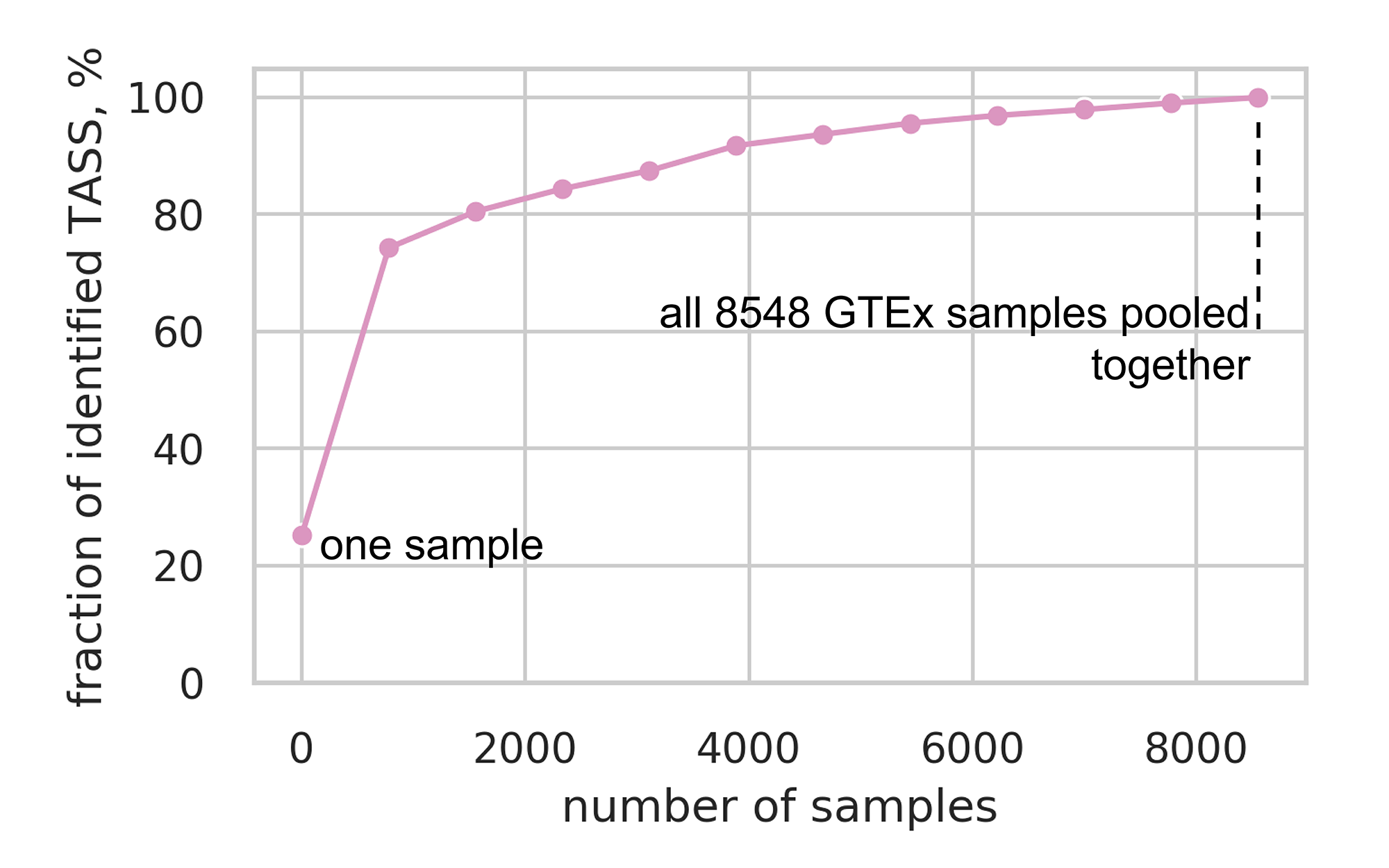

Supplement: S13 Fig — (TIF) [file pcbi.1008329.s013.tif]

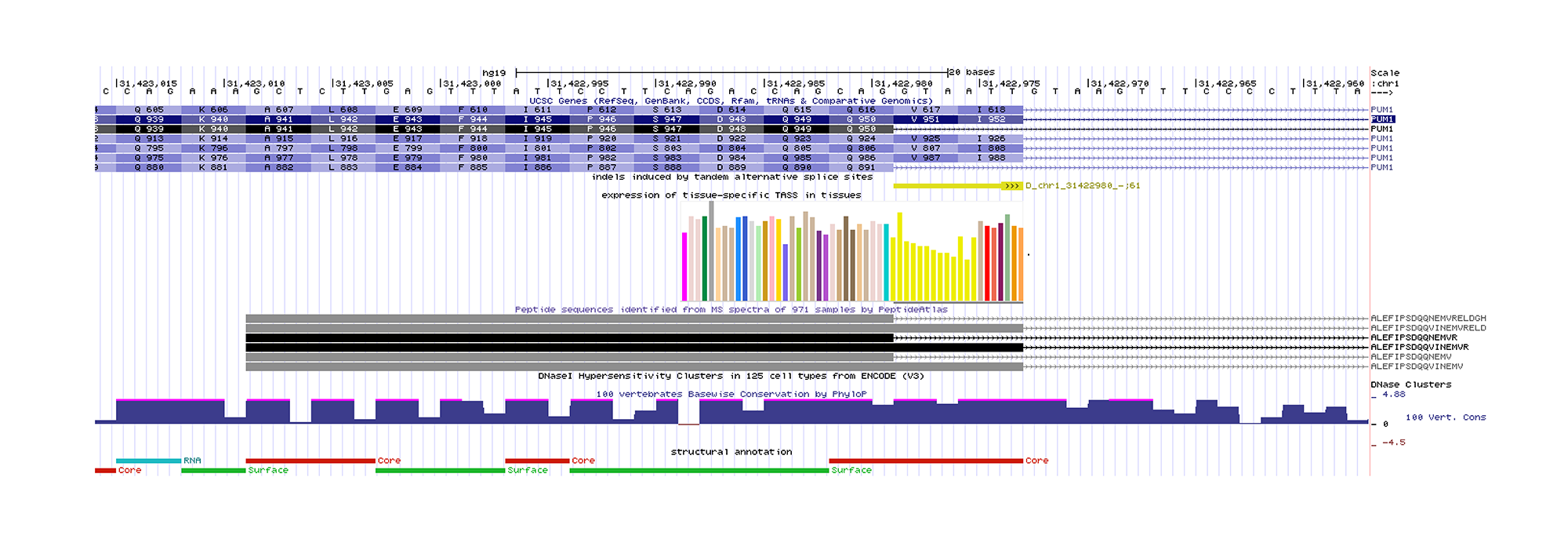

Supplement: S14 Fig — (TIF) [file pcbi.1008329.s014.tif]
