# Supplementary material for: An extended catalogue of tandem alternative splice sites in human tissue transcriptomes
Source: PLoS Comput Biol. 2021 Apr 7;17(4):e1008329. doi: 10.1371/journal.pcbi.1008329 (PMC8055015; doi:10.1371/journal.pcbi.1008329)
Supplement: Attachment — Submitted filename: TASS_paper_response_to_reviewers.pdf [file pcbi.1008329.s023.pdf]

Reviewer #1:

The study by Mironov, et al, examined the catalog of human tandem alternative splicing sites (TASS) by integrating data from multiple databases, including TASSDB2, GENCODE, UCSC, and Genotype Tissue Expression (GTEx). The authors constructed TASS clusters by grouping alternative splicing sites which are within 30nts, and in each cluster, one major splice site (maSS) was identified and the rest were regarded as minor (miSS). The author further classified the miSS TASS into different categories based on their expression patterns evaluated using GTEx data. They found that miSS with tissue-specific significant expression are conserved as maSS while the rest of miSS are much less conserved and probably full of splicing noise.

In general, the study is well designed and the analyses are reasonable. I have the following comments to hopefully improve the manuscript.

Major:

1. More explanation is needed for Fig. 3C. How was the number of co triples derived? Is this calculation controlled for the number of tissues in which a splicing event is significantly expressed?

Response: Perhaps this was not explained well in the original submission. We now specify exact conditions to call a miSS-RBP-tissue triple on page 13. For miSS-tissue pairs, the calculation was controlled for both the number of tissues and number of miSS using Q-value. In other words, the P-values obtained in the linear regression (there are “number of miSS” times “the number of tissues” such P-values) were converted to Q-values. We used the same approach for RBP-tissue pairs and miSS-RBP pairs.

2. In Fig. 4A, it seems that the authors use the group of not-significant miSS as reference, which can be a negative control. Another two refrerences such as consitutive SS and maSS can be positive controls, which represent the distributions for functional SS. Also, what is the "protein" for structure categories?

Response: Unfortunately, maSS cannot be used as a positive control since the structural category of miSS in the Fig. 4A is assigned from maSS, and the figure would be identical to that for miSS. The constitutive SS do not belong to TASS and therefore they cannot be used as any sort of control here. The “protein” label actually represents the protein-protein interaction category. Accordingly, we now use the label “PPI” instead of the “protein”. We introduce changes to the text on p. 7 l. 294 to address these questions.

3. In Fig. S11C, how was the expected value from intronic regions computed? How were the consensus sequences in intronic regions defined?

Response: We refer the reader to the original paper by Denisov et al, which describes in detail the procedure of counting substitutions at consensus nucleotides and in the control set PMID:24966225.

4. In Fig. 5D, the alphas were estimated using the Cn-to-Nc substitutions? Would the estimates from the Nc-to-Cn substitutions match?

Response: We thank the Referee for this question. The estimates from the Nc-to-Cn substitutions can in principle be computed for this dataset, but then there is a problem with the positive set because most constitutive SS have Cn nucleotides in the ancestral state. Consequently, the estimates from the Nc-to-Cn substitutions do not match the estimates from the Cn-to-Nc substitutions for not significant and significant miSS. In addition, the obs/exp values of Nc-to-Cn substitutions for both non-significant and significant miSS are not statistically discernible from one (Fig. 6A), which results in much wider confidence intervals for the estimates of splicing noise. We therefore did not pursue this analysis further. However, we insert a note in the text on p. 16 l. 677.

5. In Fig. S11D, why are the selection schemes different between human and marmoset lineages?

Response: By construction, we identify miSS in the human genome, not in the ancestral genome. That is, the procedure does not capture SS deaths, i.e. when the SS was expressed in the ancestral genome and is not expressed in the descendant genome due to the loss of consensus nucleotides. Thus, there is a systematic underrepresentation of Cn-to-Nc substitutions resulting in artificial signs of negative selection among non-significantly expressed miSS (Fig. S11D, left). In contrast, cryptic SS that were identified in the human genome by their sequence are enriched with Nc-to-Cn substitutions and thus may artificially show signs of positive selection (Fig. S11D, right). We therefore analyzed the substitutions in the marmoset lineage, where the substitutions process goes independently from that in the human lineage. We again refer the reader to the paper by Denisov et al PMID:24966225 for details. This outline is briefly described in Methods on p. 15 l. 648.

6. The supplementary tables are not attached to the pdf manuscript and no link for them.

Response: We apologize for the miscommunication. Most likely they were unattached during the submission. We now attach them to this submission and additionally provide URL links to access them through html from the Supplementary Information.

Minor:

1. provide the link for downloading decoy splice sites from reference 29, or provide more details of how to get the data.

Reponse: By request of another Referee we used another software (SpliceAI) for identification of cryptic splice sites. The procedure is described on p. 11 l. 476.

2. authors may consider using a histogram to show the distribution in Fig. 1D, because the CDF plot isn't easy to catch the information.

Response: We disagree. CDF is the best graphical display to deliver percentages, and many readers may prefer to look at a CDF to know what proportion of TASS have expression level

above a particular value. Histograms would make the figure overcrowded since we need to plot five of them at once. Boxplots are not more informative than CDF either (see below).

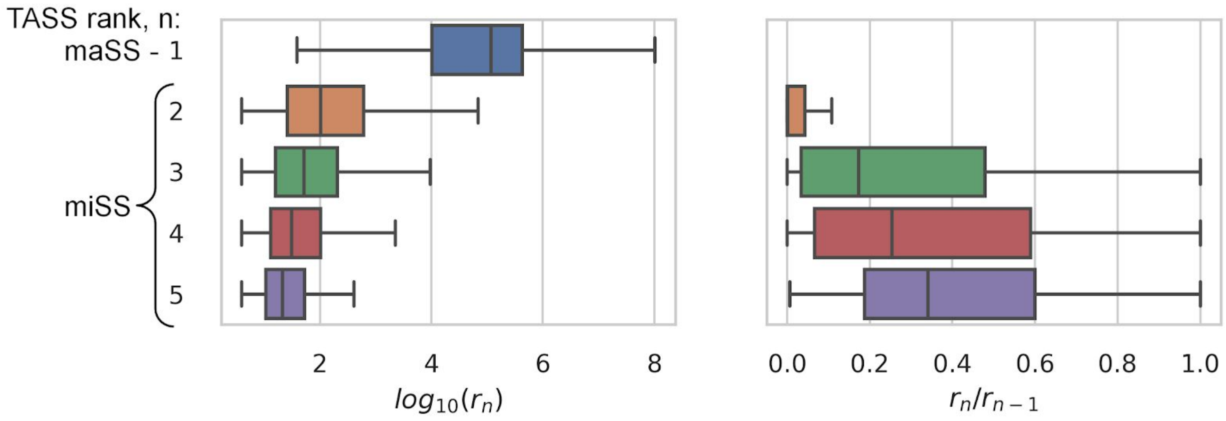

3. Page 5, line 161, Fig. 7D is referred, but there is no Fig. 7D provided.

Response. We fixed it. Is is now Fig. S6D. A change is made to the text on p. 5 l. 159.

4. In Fig. 1E, the 'coding' panel at the left, the scale for the parameter (splice site usage) should be changed to show the difference among groups.

Response: This is an expected comment, with which we strongly disagree. Changing the scale of the parameter in the left panel will give the reader a wrong impression about the magnitude of expression of miSS in comparison to the right panel. The truth is that there is almost no expression in donor sites, while in acceptor sites there is much more. We therefore insist on keeping this panel as it is now. It is shown in Fig. 1G.

Reviewer #2:

Here, Mironov et al present an updated catalog of tandem alternative splicing sites based on analyses of the data from the GTEx resource. The question about whether more than one isoform is used in most tissues is a longstanding one in the splicing field. Tandem alternative splicing sites are prevalent in the human genome and they have been associated with multiple different diseases. Even though they have been characterized previously, large volumes of data have become available over the last few years. Thus, this reviewer believes that this manuscript is a timely contribution as it is important to revisit these types of questions at regular intervals in the light of new data and findings. Overall, the manuscript is well written and the structure is logical and easy to follow. In addition to presenting genome-wide statistics, the authors also highlight multiple specific examples which I find very helpful. Nevertheless, I believe that there are several major issues that need to be addressed:

54: The authors used MaxEntScan to score putative splice sites and to discover potential novel cryptic splice sites that do not exhibit expression on the analysed RNA-seq data. They used a score threshold to only get a confident list of non-expressed cryptic sites, however it is unclear how they determined the optimal threshold.

Response: We thank the Referee for this comment. We changed our pipeline to use SpliceAI for identification of cryptic splice sites (see below).

54: Similarly, Jaganathan et. al. (Cell, 2019) have shown that SpliceAI provides significantly more specificity to predict splice sites from genomic sequence alone. The authors need to address the potentially large fraction of false positive splicing events reported by MaxEntScan by employing an alternative computational strategy, e.g. SpliceAI.

Response: We agree with the Referee and change our pipeline to use SpliceAI. Multiple changes are implemented throughout the manuscript, and the number of predictions has changed, consequently. We refer the Referee to the version with tracked changes to see the complete list of updates after changing from MaxEntScan to SpliceAI.

70: The authors say that "almost a half of the expressend splice sites are de novo". This statement is vague and the authors should provide the exact number or percentage of the total amount of expressed splice sites that were identified de novo.

Response: The information is provided in Table 1 and Table S1, which are referenced in the text on p. 3 l. 78.

Moreover, it is unclear if when they say "splice sites" they are referring to the total amount of splice sites or just the ones which support TASS. According to the Method section line 416, only 3 reads from the whole set of RNA-seq data are required to identify a splice site as expressed. Since detection of splice sites using RNA-seq is subjected to mapping errors and technical artefacts during library preparation and sequencing, it is unclear if author statement on line 70 will hold after making sure that the detected splicing events are not false positives. To reduce the number of false positives, authors do not consider novel splicing events that were flanked by annotated polymorphic sites, but this would not account for mapping errors that could be induced at lower frequency alleles. Therefore direct assessment of the mapping quality of the reads that support novel splice junctions might still be required, for example by not considering novel splice junctions that are only evidenced by reads aligned with indels around the detected splice sites. Moreover, authors could also ignore the novel splice sites that are found in only one RNA-seq sample to reduce the number of false splice site detection that is driven by experimental errors and genomic variability.

Response: In fact, in the original submission we already implemented what the Referee has suggested, namely we didn't consider novel splice junctions that are only evidenced by reads aligned with indels around the detected splice sites (see p. 11 l. 470). However, we agree with this remark and now impose more strict requirements on calling TASS from RNA-seq data (see p.11 l. 465). This resulted in a fewer number of TASS.

As for the usage of the term “splice sites” term, it refers to tandem alternative splice site when mentioned in regard of TASS, i.e., “splice sites within the TASS cluster”. Otherwise, it refers to splice sites relevant to the context.

81: Authors claim to significantly extend the number of TASS that are annotated in TASSDB2, by reporting 32,415 which are not in this database. However, this reviewer is not convinced that a larger number of splice sites is necessarily better. The authors should clarify how many of these TASSs are found when stronger criteria to avoid false positive discovery of novel splice sites are applied.

Response: We agree with the Referee in that a larger number of splice sites is not necessarily better. We now provide additional panels in Fig. 1D, 1E and Fig. S12 to exemplify the relationship between our extended catalogue and TASSDB2 and also display the expression levels of TASS in the constructed catalogue and TASSDB2. These are the only comparisons that could be made here, as the criteria in our method and in TASSDB2 differ significantly (e.g. TASSDB2 provides EST support).

81: How many of the sites found in TASSDB2 are not included in this study or not detected as expressed in the RNA-seq data?

Response: We added panels D and E to Figure 1, which contain a Venn Diagram and comparison of average expression TASS levels.

81: One of the nice features of TASSDB2 is that it has a webserver to host their data. The data presented in this study are not as accessible and the authors should ensure that it is easy for others to access to ensure that this updated catalog is used by other researchers.

Response: We disagree with the Referee. Building a separate web interface to specifically host TASS data brings in substantial development costs and operational risks related to web servers. Instead, we provide (and did provide in the original submission) a visualization tool for the TASS database through a track hub for the genome browser, which has become a golden standard in the field nowadays. For the purpose of table access, we also provide links to the tables, which are updated in real time through github.com.

146 : What is the φ distribution for the significant and non-significant TASS events?

Response: We added a panel to the Fig. 2A for those readers who might be interested in seeing this distribution directly.

152 : Authors should consider using a minimum φ value to determine if a TASS is tissue-specific. This can ensure not only a significant deviation, but also a value that could have biological relevance.

Response: Indeed, we do so (and did in the original submission) with a threshold of the minimum φ of 5%.

154 : Authors report 2,014 tissue-specific miSS. Do any of these miSS become a maSS in any of the tissues analyzed?

In the refined list we have 2,496 tissue-specific miSS. Among them, 234 (9\%) became maSS in at least one tissue. We add a comment about it in the text on p. 5 l. 153.

154: All differences reported for tissue specific miSS should be supported by numbers or supplementary figures in addition to statistical analyses.

Response: We do provide the numbers requested by the Referee in Table S2.

191 : The authors should check if these events are significantly up-regulated as a group after NMD inactivation using the data that they already analyzed in this project.

Response: We added Fig. S8E to address this comment.

213: Authors developed a statistical framework to access alternative splicing changes of TASS events after the down-regulation of different protein factors. Given that there are a variety of softwares such as DEXeq, rMATS, and Whippet that can assess alternative splicing changes of TASS as well as other types of alternative splicing events. The main limitation of the computational tools mentioned above is the need for a list of annotated splice events, generally supplied as a GTF file, limiting the analysis to annotated events. However, the authors could generate a new GTF file containing all the new splice sites discovered and use a published software to assess alternative splicing TASS changes. This will ensure the detection of robust alternative splicing changes based on a statistical framework that has been proven to have a good performance handling biological/technical RNA-seq replicates and complex alternative splicing changes. Similarly, changes in gene expression can be assessed with a diverse array of publicly available software and results of differentially expressed genes are available in ENCODE. The authors only mentioned the number of miSS-RBP-tissue triplets that they were able to detect, but they do not mention how many alternative splicing changes or gene expression changes they detected. To validate the computational strategy developed by the authors they should report how many events could be also detected using existing tools.

Response: We agree with the Referee. In the revised version we used rMATS in splice site discovery mode, which has led to several changes throughout the manuscript. We now address this in Results on p. 6 l. 210 and in Methods on p. 13 l. 525.

213: Across this section the authors analyzed a large collection of quantitative measurements derived from RNA-seq and eCLIP data. However, the details of the results are limited. For example, authors found 138 co-directed and 93 anti-directed miSS-RBP-tissue triplets, but they do not provide a figure to allow readers to visualize these changes across this data. Also, when they integrated eCLIP peak results, the authors found 7 miSS-RBP candidate pairs that are supported by eCLIP binding patterns across splice sites. However, it is unclear if the eCLIP peaks distribute differently across TASSs that are predicted to be regulated by these RBPs in comparison with all TASSs analyzed. Finally, figure 3D only shows changes relevant to one of the 7 miSS-RBP candidate pairs supported by eCLIP data across a subset from all the data analyzed. Since these analyses are highly relevant and were performed across a large set of data, authors should provide better ways to visualize the data.

Response: The procedure to obtain co-directed and anti-directed triples is shortly exemplified in Figures 3A and 3B. A figure to visualize these triples across all data, even if possible, would be three-dimensional as there are three covariates in the data (TASS, tissue, and shRNA-KD). We show projections of such a figure in the panel E of the main Figure 3 for QKI and PTBP1. We additionally provide Supplementary Table S6 summarizing the data on co-directed and anti-directed triples.

Regarding the distribution of eCLIP peaks, we show in Figure 3D that co-directed triples dominate among TASS with eCLIP peaks; however, this positive association for a 2x2 contingency table implies that eCLIP peaks are also enriched within co-directed than anti-directed triples. There is no room in Figure 3 to demonstrate the distribution of eCLIP peaks.

Regarding the comment about better ways to visualize the data, we once again note that the summary of all the observed changes for all miSS would require a three-dimensional display, which is impractical. The boxplots shown in Figure 3E sufficiently well describe the relationship between TASS and RBP expression for one particular example.

264 : The statement “The residues in this part of the helix become more hydrophobic, which may influence the overall helix or protein stability” is currently not supported by quantitative analyses. Given the array of different available computational tools that can be used to assess protein stability and structure prediction of protein domains, authors should perform a quantitative analysis to suggest this or at least cite relevant literature to backup this claim.

Response: We have analyzed the stability of the two isoforms with FoldX, which is, to the best of our knowledge, the only tool that allows to easily assess the overall protein stability (as opposed to a multitude of tools to analyze the change of stability upon point mutations). An alternative to that could be performing a molecular dynamics simulation and assessing the free folding energy from it, but it is a very resource-consuming procedure and lies far beyond the scope of this study. The corresponding changes are on p. 8 l . 303.

Overall this manuscript lacks substantial biological novelty beyond additional events being detected and identification of possible regulatory RBPs associated to TASSs. To gain further biological insight authors could for example try to further analyze the genomic variants associated to quantitative miSS changes or explore how TASS is regulated through cell-types using publicly available TRAP-seq or scRNA-seq data.

Response: The answer to this comment is twofold.

The analysis of genomic variants associated with quantitative miSS changes within the GTEx dataset is strongly confounded by the procedure in which these miSS were found: one needs to exclude the genomic variants that may interfere with short read mapping. We therefore chose not to perform this analysis as it could be misleading for the reader. Instead, we analysed the allelic frequencies associated with tissue-specific and non-tissue-specific miSS, which are now presented in Fig. 6B. This is perhaps the furthest we could go in following Referee’s comment about genomic variants.

Regarding the analysis of since cell RNA-seq, we note that the inherent sparsity of these data precludes reliable detection of miSS, which are already sparse at the scale of GTEx RNA-seq panel, the largest compendium of human tissue RNA-seqs available to date. To address this comment, we chose to analyze a recently published panel of RNA-seq experiments in human primary cells from PMID:32759341. We added a new figure (Fig. 4), in which we show the intersection miSS derived from GTEx with miSS derived from PROMO cells and analyse cell-type specific and tissue-of-origin-specific expression of miSS in these cells.

We disagree with the Referee in that our manuscript lacks substantial biological novelty: The examples presented in Figures 2D (NPTN), 3E (QKI), 4C and 4D (IGFLR1 and RBM42), figures 5E,F,G (PICALM, PUM1, ANAPC5) as well as examples shown in supplementary figures do provide substantial novel insights about TASS splicing and its regulation. Further analysis going beyond these examples could be a topic of a more specialized research, and exactly by this reason we present our TASS catalog to allow further investigation on this topic.

I have also identified the following minor issues:

50: MIn is not a commonly used abbreviation in the literature and it is not currently defined here.

Response: We agree. This is fixed.

67: Authors should give the exact number of splice sites and express the numbers corresponding to each category as an approximated percentage.

Response: For readers' convenience, the exact numbers are provided in Supplementary Tables, while in the main text we round values to thousands. We added the percentages on p. 3 l. 69 as requested.

70: In this context an exon could be cataloged as "non-coding" for proteins located at UTRs or genes which do not code for proteins. The numbers would be more clear if authors provide separate numbers for TASS that belong to UTRs or non-coding genes.

Response: We added the requested information in Fig. S1

78: This is expected, however authors have not explained which expression unit they are using. Does Rn just refer to the number of reads? In the case Rn corresponds to the number of reads, it would be better to use an expression unit that is not confounded by gene expression, such as PSI or the alternative metric the authors introduced.

Response: Rn indeed denotes the number of reads. It is a widely used metric that gives the reader an idea of the absolute support of TASS expression. If the Referee is interested in the relative expression, right next to the distribution of Rn on Fig. 1F we plot the distribution of $r_n/(r_1+r_n)$, which addresses this comment.

Table 1: The two bottom columns from `% of split reads supporting TASS` column should add up to 100? The sum of the numbers provided by the authors is 100.1.

Response: This is now corrected.

85: Authors should carefully check for grammar mistakes, for example here “from the split reads” should just be “from split reads”.

Response: We are doing our best with the grammar, however we cannot guarantee 100% correct use of definite articles. If this paper is accepted for publication, we will kindly ask the Editorial Office for a proofread by a native English speaker.

86: “Indeed, we checked that only 2.2% of split reads that support miSS on one end support several splice sites on the other end” is not very understandable. I was expecting you to report the percentage of novel splice junctions that were in your data which “neither donor nor acceptor splice site is annotated”.

Response: We agree that this sentence is awkward. We meant to say that only a small fraction of split reads supporting miSS on one end of the junction land on several splice sites on its other end. This sentence is not important and is now removed.

124: “..while miSS located upstream tend to be expressed stronger than miSS located downstream “. Is an interesting claim that should be backed up by statistical analyses.

Response: We added an additional panel, Fig. S4B.

128 : Statistical analyses are missing.

We updated Fig. S5A

129 : Statistical analyses are missing.

We updated Fig. S5B

268 : Does an alternative acceptors that are 39 nt apart still count as tandem alternative spice stites? Which is the maximum distance at which alternative splicing 5’/3’ splicing events are considered as TASS?

Response: According to our definition, a cluster of TASS is defined as a series of splice sites of the same type (donor or acceptor) that are separated by not more than 30 nts between each pair of consecutive splice sites. Therefore, the distance between the first and the last TASS in a cluster can be more than 30 nts if there is another TASS in between. This is the case for the TASS cluster shown in Fig. 5G. We add a comment to the figure caption to clarify this.

296 : Authors should report the p-value and statistical test utilized to assess corresponding statistical significance.

Response: The statistical method used to estimate the strength of the evolutionary selection acting on splice sites was taken from the paper by Denisov et al, which is referenced in the beginning of the section. In brief, it constructs confidence intervals for the ratio of two binomial proportions based on likelihood scores. The significance of the differences can be inferred from the confidence intervals, and therefore there is no need for additionally crowding the figure with

p-values. However, we agree to add a citation to the original 1995 paper by Nam et al on p. 8 l. 325.

309 : The statement “we observed only a subtle difference in evolutionary selection between” it is vague. Authors should report the magnitude of the difference and some parameters to claim these are just subtle differences.

Response: We agree that this statement is vague. We reformulate it as “We observed no significant difference in evolutionary selection..” and move the corresponding panel to the supplementary information (Figure S11F). The absence of significant difference in evolutionary selection is evidenced by overlapping confidence intervals.

Figure 1E: Colours need to be explained.

Response: We agree. The red and the blue bars denote frame-disrupting and frame-preserving shifts. We added a legend to the figure.

Figure 1F: The significance should be coded by *, **, *** marks. The exact p-value and statistical test should be included in the figure legends or main text.

Response: We agree, this is corrected.

Figure 2E: This figure should be wider.

Response: We agree, this is corrected.

Figure 4A-D and Figure 5A-B: Authors should explain the meaning of the error bars and highlight any statistical difference found while comparing these measurements.

Response: We agree that this is unclear. The error bars in 4A-D (5A-D in the revised version) correspond to a 1-sample proportion test. The error bars in Figure 5A (6A in the revised version) correspond to confidence limits for the ratio of two binomial proportions based on likelihood scores, as explained earlier. We add the explanation to the figure legends.

Reviewer #3:

Mironov et al. present a new, more comprehensive catalogue of human TASS cases thas has been compiled based on recent RNA-seq data. In my view, this alone has only minor impact for the research field. More interesting is the investigation on tissue specificity of TASS isoform ratios. However, presentation of this latter part is quite condensed and difficult to track. The manuscript should be improved to clarify the specific outcome of the analysis and correctly put it into context of the heterogenous TASS catalogue. This is particularly important for the proposed mechanism of PTB acting as a tissue-specific regulator of TASS isoform formation.

MAJOR

1. The authors claim that they "substantially extend the existing catalogue of TASS" (l. 37), which is probably correct. The significance of this progress should be analyzed with respect to

significance of TASS outcome. TASS isoform products are the more likely to be functionally relevant the more balanced the isoform ratios (high phi values) are. One can speculate that TASS cases identified in this study are the ones with very low miSS (less likely to be functionally relevant) because this would be an explanation why previous studies (using less sequence data) have overlooked these cases. The authors should analyze the phi value in the newly identified TASS cases in relation to previously known cases.

Response: We added a supplementary figure (Fig. S12B) demonstrating that miSS that are present in TASSDB2 have on average lower phi values than miSS from TASSDB2. However, we also show that the newfound miSS are enriched in tissue-specific and significantly expressed categories, and such miSS tend to have similar or higher phi values compared to miSS in TASSDB2 (Fig. S12C). Besides this, there are a substantial number of novel significantly expressed NAGNAGs (1032 in total, including 190 tissue-specific ones) and novel significantly expressed GYNNGYs (356 in total, including 37 tissue-specific ones), see Fig S8A and S8C. These data confirm at a large proportion of newly identified miSS are functionally important.

Regarding the comment that TASS in this study may less likely be functionally relevant, we note that there has been no large-scale study of RNA-seq data devoted to TASS, and clearly the analysis of this data source as compared to ESTs in TASSBD2 is a major improvement in our study. Lower expression level of some TASS does not invalidate it, but rather makes it more interesting to discover novel TASS at higher depth and resolution.

2. Important previous studies on tissue-specific TASS are not cited and not discussed: DOIs 10.1101/gr.186783.114 and 10.1093/nar/gku532, as far as i oversee. These must be included in a general outline on models of TASS regulation.

Response: We thank the Referee for these references. We now cite them on p. 6 l. 243.

3. When it comes to functional characteristics, esp. tissue-specificity of splicing, Mironov et al. hardly differentiate the TASS subtypes. Only the NAGNAG subtype (acceptors in 3 nt distance), probably the largest subgroup, is analyzed separately. 429 of 7414 NAGNAG cases (5.8%) appear to be less frequently tissue-specific compared to TASS average, 2014 of 12361 (16.3%). Tandem donors form another specific subtype, which deserve specific consideration. A separation of the subtypes offer important mechanistic insights. Splice site distance is another relevant structural property - see next point.

Response: Perhaps the Referee has overlooked the analysis of GYNNGY donor splice sites, which is another separate group (page 5 l. 191). In other analyses, we agree that donor and acceptor splice sites have to be discussed separately. We do analyse the shift distributions of donor and acceptor splice sites separately on Figure 1G, and introduce an extra panel in supplementary Figure S6. However, it was not our goal per se to characterize different TASS subclasses beyond overlapping consensus sequences. Since we analyze splice site distance (aka shift) in Figures 1G, we therefore leave this more specific analysis to separate future studies.

4. How general is the proposed mechanism of PTBP1 acting as a tissue-specific regulator of TASS isoform formation? This is an important question. I suppose, and this should definitely be tested, that a regulatory involvement of PTB in tissue-specific splicing is positively associated with splice site distance. This is likely because PTB binds to the polypyrimidine tract; a polypyrimidine tract overlapping the TASS region, the longer the more efficient, would be a plausible action platform for PTB interference.

Response: We thank the Referee for this excellent question. We implemented the analysis suggested by the Referee and it turned out to be exactly as he has guessed. We included additional panels in Fig 3 (Fig 3F and Fig 3G) and amended the text on p. 6 l. 239.

5. What is the fraction of RBP association with tissue-specific TASS that is explained by PTB (or other particular factor)? The fraction of PTBP1-associated tissue-specific TASS in total? This would hint to unexplored contributors of tissue-specificity. The supplement announces table data (I cannot inspect) towards this question but, anyway, these general questions need to be addressed in the main text.

Response: This is an interesting question; however, we have only 163 cases of miSS regulation by RBP that is concordant between tissue analysis and shRNA-KD of the RBP. Small size of this set precludes any rigorous analysis of variance decomposition for PTBP1 or any other factor (if we understand well what Referee had in mind). However, given the interest of the readership in PTBP1 in particular, we performed additional analysis of PTBP1 overexpression data. Figure 3 now has two additional panels, 3F and 3G. All supplementary tables have been updated.

6. Fig. 2, panel A is meant to illustrate evidence for alternative splicing of TASS cases. However, highlighting of NPTN (tissue-specific TASS example) suggests it might illustrate tissue-specific TASS splicing. To make the steps fully clear, I suggest to place panel B as panel A; panel A as panel B omitting the NPTN highlight and add an additional panel (neo)C which shows the separation of tissue-specific and non-tissue-specific TASS cases with the NPTN highlight. QKI, the tissue-specific example illustrated in fig. 3, must also be highlighted.

Response: We thank the referee for this suggestion. However, exchanging panels (A) and (B) in Fig 2. will break the logic of the presentation since we first introduce ZIP regression to identify significantly expressed TASS in (A) and only after that we show the split up of significantly expressed TASS into tissue-specific and non-tissue-specific TASS in (B). However, we do follow the suggestion of the Referee to unlabel NPTN in Fig 2A. We also add a panel demonstrating that tissue-specific miSS are separated from non-tissue-specific miSS by Q-value<0.05 and the estimated Delta phi value more than 0.05 (Fig S6A). In regard to Fig 2C, we comment that the entire panel 2C is about NPTN (the axes in Fig 2C are different from those in Fig. 2A). Therefore the suggestion to show an example of a TASS without tissue-specific separation will involve another panel. We provide this extra panel as Supplementary Figures 7A and 7B in order to avoid overcrowding of Fig 2.

7. In the methods to detect regulation of miSS by RBP (l. 476 ff), how is the background distribution of the slope modeled? This should be specified. Same for tissue-specific miSS (l. 456 ff).

Response: By request of another Referee we changed miSS analysis in shRNA-KD of RBP to rMATS, a standard tool in the field. In regard of tissue-specific miSS, the background distribution of the slope was modelled within the linear model of quantile regression. For detailed information we refer the reader to Methods p. 12 l. 505 and p. 13 l. 525.

MINOR

8. Reference to cystic fibrosis as a severe disease caused by single-aa indel [11] (l. 15) in the context of TASS is misleading because the variant is a mutation, which is subject to purifying selection (although balaced by minor advantageous effects). In contrast, TASS generates isoform molecules from the same allele, likely have passed purifying selection (esp. with equi-expressed isoforms), may be even subject to positive selection (gain of function). This reference should be omitted or made clear by explanation.

Response: We agree to omit this reference.

9. The statistics for miSS expression has a flaw in correcting for multiple testing. As the authors state (l. 449), multiple testing is corrected by a Q-value metric at the level of individual tissue. However, in the analysis of multiple tissues the Q-value metric is no longer valid to describe the meta-significance appropriately. The metric needs to be adjusted to nested multiple testing.

Response: We apologize for incorrectly stating this procedure in Methods. We actually control for both the number of tissues and number of miSS using Q-value. In other words, the P-values obtained with ZIP regression modelling (there are “number of miSS” times “the number of tissues” such P-values) were converted to Q-values. Therefore, there is no flaw in our analysis. Furthermore, the procedure we present provides the most pessimistic estimate of the statistical significance. Accordingly, we corrected the text on p. 12, l. 501.

10. What is the straight line Fig. 2A representing? Apparently, it is not relevant for separation of significant and non-significant miSS (tracability of minor isoform).

Response: Perhaps the legend didn’t phrase this well. The solid curve in Fig 2A corresponds to the fitted value of the parameter of ZIP regression, while the dotted curve corresponds to the FDR cutoff of 5%. These are not straight lines. Additional details have been added to the legend, and the figure has been reworked to show that these lines are not straight.
