# Supplementary material for: An extended catalogue of tandem alternative splice sites in human tissue transcriptomes
Source: PLoS Comput Biol. 2021 Apr 7;17(4):e1008329. doi: 10.1371/journal.pcbi.1008329 (PMC8055015; doi:10.1371/journal.pcbi.1008329)
Supplement: Attachment — Submitted filename: response_to_referees.pdf [file pcbi.1008329.s024.pdf]

Reviewer #1:

By incorporating the comments from the reviewers, the manuscript has been improved in clarity and reliability. The authors have addressed most of my concerns, and here are a few remaining things needing authors' inputs:

1. In Fig 5A (4A in old version), I proposed to show the structure category distribution for maSS and constitutive splicing sites, because they represent the distributions for functional splicing sites. Will the significant expressed tissue-specific miSS (supposed to be functional) have a similar distribution with those distributions?

Response: To address this point, in the previous round of revision we added Figure S10A, which describes structural categories of miSS and maSS. We agree with the Referee that the distribution of structural categories of constitutive splice sites may interest readers and therefore we add to Fig 5A a series of bars that correspond to constitutive splice sites. However, structural categories of constitutive splice sites are most similar to those of non-significant miSS, not tissue-specific miSS. This happens because a miSS's structural category is inferred from that of its maSS, and therefore non-significant miSS, in which there is not much of alternative splicing, mirror the background frequencies of structural categories of all splice sites, the majority of which are constitutive. A short comment about it is added on p. 7 l. 275.

2. Page 3, Line 56, it states that there are ~600K cryptic sites, but Table S1A shows only 196885 sites. Please explain it.

Response: We thank the Referee for this correction. Indeed, the correct number is 196,885 sites. We modified the text on p. 3 l. 58

Reviewer #2:

Overall I think the authors have improved the manuscript significantly and they have addressed all of my comments. Nevertheless, there are some minor issues remaining.

1) they said that in the original submission they took care of reads aligning with miss matches around splice sites, but they just mentioned filtering steps related with annotated polymorphisms.

Response: In the previous revision, we explain it in methods on p. 11 l. 474. In this revised version, we additionally mention in the results section on p. 3 l. 51 that we applied filtering steps to control for split read misalignments that are caused by the presence of germline polymorphisms near splice sites (see Materials and Methods for details). The manuscript was amended on p. 3 l. 51.

2) I agree with the authors that making the data available as UCSC hub, however neither within the tracks or in the manuscript there I could find documentation that enables me to fully interpret the data displayed on these tracks (e.g. explanation of the different colors).

Response: We thank the Referee for this comment. We modified the presentation of the track hub to have all tracks in the “pack” mode, in which a user can immediately see the structural categories and their corresponding colors. We also added a description URL within the track hub for user’s convenience.

3) even though it might not be author’s fault, the figure quality on this submission was very low, making it quite hard to interpret the results.

Response: All figures are now provided in high quality in .TIF format. We additionally worked out the figures using Preflight Analysis and Conversion Engine (PACE), as suggested.

Reviewer #3:

All my remarks have been addressed properly.
